# Suicidal Ideation among Youth Living in the Slums of Kampala, Uganda

**DOI:** 10.3390/ijerph15020298

**Published:** 2018-02-09

**Authors:** Rachel Culbreth, Monica H. Swahn, David Ndetei, Lynnette Ametewee, Rogers Kasirye

**Affiliations:** 1 School of Public Health, Georgia State University, P.O. Box 3984, Atlanta, GA 30302, USA; mswahn@gsu.edu (M.H.S.); lametewee@gmail.com (L.A.); 2 Department of Psychiatry, University of Nairobi, University Way, Nairobi 00100, Kenya; dmndetei@amhf.or.ke; 3 Africa Mental Health Foundation, P.O. Box 48423-00100, Nairobi 00100, Kenya; 4 Uganda Youth Development Link, Sir Apollo Kaggwa Rd, P.O. Box 12659, Kampala 00256, Uganda; kasiryer@yahoo.com

**Keywords:** adolescent health, suicidal ideation, problem drinking, high-risk youth, sub-Saharan Africa

## Abstract

The purpose of this study is to examine the factors associated with suicidal ideation among youth living in the slums of Kampala, Uganda. Analyses are based on cross-sectional survey data, collected in 2014, of a convenience sample (*n* = 1134) of urban service-seeking youth participating in a Uganda Youth Development Link drop-in center. Logistic regression analyses were computed to determine the psychosocial factors associated with suicidal ideation. Among youth participants, 23.54% (*n* = 266) reported suicidal ideation in the past year. In the multivariable analysis, suicidal ideation was associated with being female (OR: 1.61; 95% CI: 1.15, 2.25), reporting one (OR: 1.51; 95% CI: 1.05, 2.18) or two deceased parents (OR: 1.55; 95% CI: 1.03, 2.35), ever living on the streets (OR: 2.65; 95% CI: 1.86, 3.79), problem drinking (OR: 1.83; 95% CI: 1.19, 2.80), sexually transmitted infection (OR: 1.59; 95% CI: 1.14, 2.21), ever being raped (OR: 1.49; 95% CI: 1.01, 2.20), and experiencing physical child abuse (OR: 2.40; 95% CI: 1.75, 3.27). Our findings underscore many unmet needs in this vulnerable population. However, strategies that specifically seek to address problem drinking—a modifiable risk factor for suicidal ideation—may be particularly warranted in this low-resource setting.

## 1. Introduction

Suicide is a major public health problem worldwide and the third leading cause of death for adolescents ages 15–19 [1]. While there is a scarcity of literature on suicidality and associated risk factors in sub-Saharan Africa, there is a growing recognition of the problem and also of suicidal ideation as a pressing problem [2]. To-date, most of the previous literature has focused on suicidal behavior and ideation in high-income Western countries [2]. As an example, in the United States, an estimated 17.0% of high-school youth have reported suicidal ideation in the past year [3], whereas reports of suicidal ideation among youth in Uganda are much higher (27.9%) [4]. Moreover, youth living in the slums or on the streets may be at an even higher risk for both suicidal behaviors and ideation, which may be exacerbated by their dire environmental conditions [5,6,7,8,9]. A previous but small study documented a 30.6% prevalence of suicidal ideation among youth living in the slums of Kampala, Uganda [5]. 

While there may be unique risk factors for suicidal behaviors and death among youth in sub-Saharan Africa, there are likely also similarities to those of adolescents in other regions of the world. Previous research shows that living on the streets [10], substance use [2,5,11,12,13,14,15,16], adverse childhood experiences [11,17,18], and HIV/AIDS [17] and sexually transmitted infections (STIs) [5,19,20] are important risk factors for suicidality among youth. HIV/AIDS has been linked to depressive disorder and psychological distress, potentially leading to suicidal ideation in adolescents [21,22,23,24]. Ng and colleagues reported a 20% prevalence of suicidal ideation among HIV-positive youth living in Rwanda—much higher than the 12% prevalence among HIV-negative children [17]. Additionally, acquiring an STI has been associated with psychological distress and suicidal ideation [5,19,20]. Moreover, adverse childhood experiences, including experiencing child abuse (perpetrated by parents or caregivers) and experiencing childhood rape, have been well established in the literature as having a direct impact on depression, suicidal ideation, and suicidal behaviors [11,17,18]. Mechanisms of association between adverse childhood experiences include biological mechanisms through which repeated toxic stress may disrupt brain development and certain brain structures, leading to stress-related diseases and cognitive impairment [25]. These impairments and traumatic residual effects have been linked to an increased risk of suicidal ideation and suicidal behavior [11,17,18]. Substance use—in particular alcohol use and misuse such as problem drinking and drunkenness—has also been well-established in the literature as having an association with suicidal ideation and attempt among adolescents [11,12,13,14,15,16]; however, few studies have examined these associations in sub-Saharan Africa [2,5]. 

In Uganda, suicidal ideation and behaviors have been identified as an emerging public health problem among adolescents [2], particularly among youth in dire environmental conditions such as youth living on the streets and slums of Kampala, Uganda [5]. Some of the unique vulnerabilities of youth in the slums are related to living in absolute poverty without adequate access to clean water, electricity, and sanitation, and also exposure to high levels of crime including violent victimizations. Slum communities are typically characterized by their lack of basic infrastructure, government resources, and social services. Additionally, slums consist of a broad range of poor environmental, social, and economic conditions [26] which likely exacerbate the mental health conditions of vulnerable youth who experience a multitude of other risk factors and adversity. 

To add to the limited research on suicidal ideation among youth in slums, the current study examines the psychosocial risk factors for suicidal ideation in a relatively large cross-sectional study of youth living in the slums of Kampala (*n* = 1134). This study expands on the research presented previously from a smaller study, also conducted in the slums of Kampala in 2011 [5], by examining the risk factors for suicide ideation in a much larger sample—a sample with a narrower age range and with a higher proportion of males. The current analyses focus on key demographic characteristics and psychosocial factors, several of which were not addressed in the previous study (i.e., age, gender, education, ever living on the streets, parental living status, alcohol and problem drinking, HIV infection, STI infection, ever being raped, and parental physical abuse of the youth). Our hypothesis is that living on the streets, problem drinking, HIV and STI infection, previously being raped, and physical child abuse will be associated with suicidal ideation. The goal of this research is to provide new empirical data on suicide ideation among vulnerable youth in the slums while also providing information that can guide service provision and the development of interventions for youth in low-resource settings.

## 2. Materials and Methods

### 2.1. Setting

The current study is based on the “Kampala Youth Survey 2014”, a cross-sectional survey conducted in the spring of 2014 to quantify and examine high-risk behaviors, with a focus on alcohol use, sexual behaviors, and HIV. The sample consisted of urban service-seeking youth, 12–18 years of age, living in the slums or on the streets of Kampala, Uganda, who were participating in a Uganda Youth Development Link (UYDEL) drop-in center for disadvantaged street and slum youth [27]. Study participants were recruited at six drop-in centers and the neighborhoods surrounding the UYDEL drop-in centers primarily through word-of-mouth.

### 2.2. Data Collection

Survey data collection methodology is mentioned in a previous paper [28]; however, the final sample size yielded 1134 youth participants (ages 12–18) with completed surveys. Participants were informed about the study and read (or were read) the consent forms for the survey. All participants provided verbal consent to participate in the study. Youth who “cater for their own livelihood” are considered emancipated in Uganda and are able to provide their own consent for the survey without parental consent. Participation was limited to youth ages 12–18 present in-person on the day of the field visit. There were no other exclusion criteria. All subjects gave their informed consent for inclusion before they participated in the study. The study was conducted in accordance with the Declaration of Helsinki, and IRB approvals were obtained from Georgia State University and the Uganda National Council for Science and Technology to conduct this study in Kampala (SS3338). 

The Kampala Youth Survey 2014 included questions on alcohol use, alcohol marketing, sexual risk behaviors, adverse experiences, gender-based violence, and general violence. The survey questions were utilized from previously validated measures, including the Global School-based Student Health Survey (GSHS) [29], Kampala Youth Survey 2011 [5,6,7,28,30], MAMPA 2012 Questionnaire, AUDIT Questionnaire [31], CAGE Questionnaire [32], iMPPACS, AIDS Indicator Survey [33], and the Demographic Health Survey [34].

### 2.3. Measures

The main outcome variable—suicidal ideation—was measured using the following question, “In the past year, did you ever think of killing yourself?” Respondents could answer “Yes” or “No”. Sociodemographic variables included gender (male or female), age, parental living status (both parents dead, one parent dead, both parents living), and education (primary education or less, completed primary education, and secondary education or higher). Hypothesized risk factors were ascertained from previous studies and the growing body of literature on suicidal ideation among youth in developing countries. Problematic alcohol use was operationalized using the CAGE questionnaire [32]. The CAGE questionnaire consists of four questions, “Have you ever felt you should cut down on your drinking?”; “Have people annoyed you by criticizing your drinking?”; “Have you ever felt bad or guilty about your drinking?”; and “Have you ever had a drink first thing in the morning to steady your nerves or to get rid of a hangover (eye opener)?” Participants could answer yes (1) or no (0). CAGE scores were totaled, and scores of 2 or greater indicate problem drinking, whereas scores lower than 2 indicate non-problem drinking. For the purpose of this analysis, the variable was categorized into problem drinkers, non-problem drinkers, and non-drinkers. HIV infection and STIs were self-reported. Previously being raped was measured using the following question, “Has someone ever raped you or forced you to have sex with him or her?” Experiencing childhood physical abuse perpetrated by a parent was measured using the following question, “Did your parents ever beat you so hard you had bruises or marks?” Respondents could answer “Yes” or “No” to all questions.

### 2.4. Data Analysis

Descriptive statistics were computed for suicidal ideation. Descriptive statistics were also computed for sociodemographic variables and hypothesized risk factors among outcome categories (suicidal ideation vs. no suicidal ideation). Bivariate and multivariable logistic regression analyses were used to determine the association between suicidal ideation and the hypothesized risk factors, adjusting for sociodemographic variables. All analyses were performed using SAS 9.4 (SAS Institute, Cary, NC, USA).

## 3. Results

Among the youth participants (*n* = 1130), 266 (23.54%) reported suicidal ideation in the past year (Table 1). While the overall sample was comprised of mostly females (56.24%), a larger percentage of females reported suicidal ideation (64.66%) compared to the total sample. A much higher percentage of youth who reported suicidal ideation also reported ever living on the streets (39.47%) compared to youth who did not report suicidal ideation (16.57%). Most of the youth who reported suicidal ideation also reported one or two deceased parents (73.31%) compared to just over half of the youth who did not report suicidal ideation (55.33%). Nearly half of youth who reported suicidal ideation also reported alcohol use (48.12%), which was nearly twice as high compared to non-suicidal youth (25.00%). Additionally, a higher percentage of suicidal youth reported HIV infection (13.91%), STIs (53.38%), ever being raped (28.95%), and experiencing childhood abuse perpetrated by a parent (51.50%). 

In the bivariate analyses (Table 2), older ages were associated with suicidal ideation (OR: 1.12; 95% CI: 1.03, 1.22) and being female (OR: 1.58; 95% CI: 1.19, 2.10). Reporting one parent (OR: 2.08; 95% CI: 1.49, 2.89) and both parents dead (OR: 2.47; 95% CI: 1.71, 3.56) were significantly associated with suicidal ideation. Additionally, ever living on the streets (OR: 3.28; 95% CI: 2.42, 4.45), problem drinking (OR: 3.50; 95% CI: 2.44, 5.01), HIV infection (OR: 1.61; 95% CI: 1.06, 2.44), STI infection (OR: 2.43; 95% CI: 1.83, 3.21), ever being raped (OR: 2.68; 95% CI: 1.92, 3.72), and experiencing parental abuse (OR: 2.71; 95% CI: 2.04, 3.59) were all statistically associated with suicidal ideation in the bivariate analyses.

In the multivariable analyses, being female (OR: 1.61; 95% CI: 1.15, 2.25), ever living on the streets (OR: 2.65; 95% CI: 1.86, 3.79), reporting one parent (OR: 1.51; 95% CI: 1.05, 2.18) or reporting both parents dead (OR: 1.55; 95% CI: 1.03, 2.35), problem drinking (OR: 1.83; 95% CI: 1.19, 2.80), STI infection (OR: 1.59; 95% CI: 1.14, 2.21), ever being raped (OR: 1.49; 95% CI: 1.01, 2.20), and experiencing parental physical abuse (OR: 2.40; 95% CI: 1.75, 3.27) were all statistically associated with suicidal ideation. HIV infection, education, and age were no longer statistically associated with suicidal ideation in the multivariable analysis.

## 4. Discussion

Nearly one-fourth of the youth in the current study reported suicidal ideation in the past year. The findings support previous research by demonstrating that youth who reported a range of adverse experiences such as having lived on the street, having one or two deceased parents, physical abuse and rape were significantly more likely to also report suicidal ideation. Moreover, youth who reported having an STI and who identified as a problem drinker on the CAGE screening tool were also more likely to report suicidal ideation. 

The prevalence of suicidal ideation in this study was slightly higher than the estimates of both suicidal ideation among United States adolescents (17.0%) [3] and among nationally representative school-attending youth in Uganda (19.6%) [4]. However, the prevalence of suicidal ideation in the current study is lower than the prevalence of suicidal ideation in a previous study of youth living in the slums of Kampala (30.6%) [5]. The previous study, conducted in 2011, consisted of a smaller sample of youth and also included older youth [5] which might explain the differences between the two studies.

Contrary to previous literature on the association between HIV/AIDS and suicidal ideation [5,17], we failed to detect a statistically significant association in the multivariable analysis. However, we found that having an STI was associated with suicidal ideation in the multivariable analyses. A possible but potentially unsatisfactory explanation for the lack of a significant association between HIV and suicidal ideation which we had hypothesized in this study may be that these youth experience other more pressing psychosocial stressors and adverse childhood experiences (e.g., homelessness, physical abuse, and rape) that impact their life, which we controlled for in our multivariable analyses. Moreover, the youth in our study could have contracted HIV at birth and not necessarily through sexual activity (unlike having other sexually transmitted STIs). The method of HIV acquisition (e.g., through birth, rape, or unprotected sex) may be an important factor in the association between HIV and mental health concerns such as depression and suicidal ideation that we cannot examine in the current survey but would be an important factor to consider in future research. An alternate explanation may be that an HIV diagnosis may not be as psychologically burdensome for these service-seeking youth due to the availability of anti-retroviral medication [35] and psychosocial support offered at the drop-in center—another factor we cannot specifically examine with the available data but which needs to be addressed in future research. 

One of the key findings in our study supports previous research [2,5,11,12,13,14,15,16] by demonstrating a strong association between problem drinking and suicidal ideation. In this study, problem drinking was assessed using the CAGE screening tool. Those youth who reporting problem drinking were 1.83 times more likely to report suicidal ideation in multivariable analyses compared to youth who did not drink. Problem drinking is likely a coping strategy for these youth with high levels of adverse childhood experiences (e.g., physical abuse, being an orphan, homelessness, and rape) and current health issues HIV/AIDS and other STIs. However, of the factors examined in this study, problem drinking is a modifiable risk factor for suicidal ideation that could potentially be replaced with other coping strategies and psychosocial support that may address the underlying reasons for alcohol misuse. It should be noted here that most of the youth in this survey were under the legal drinking age of 18 years in Uganda. As such, strategies that seek to address underage drinking and limit availability among minors may in turn also result in lower levels of problem drinking in this vulnerable population. Additionally, it is important to also consider the context of the living environment in the slums. These youth have limited access to social services and government resources and often face poor environmental, social, and economic conditions that likely exacerbate their existing health conditions and adverse childhood experiences. As evident from the findings from this study, these youth face a multitude of hardships beyond their dire living conditions that may predispose them to suicidal ideation and problem drinking. 

### 4.1. Limitations

There are several limitations that should be considered when interpreting the findings from this study. Suicidal ideation as well as all the other psychosocial factors examined in this study were self-reported and may be subject to recall, response, and social desirability bias. This survey of service-seeking youth also comprised a convenience sample, which may limit the generalizability of the results. It should be noted, however, that this population is particularly difficult to reach, and convenience sampling represents a commonly used methodology for research on this and similar populations [5,6,7,28,30]. 

### 4.2. Implications

Despite the noted limitations, this study has multiple strengths. Data on suicidal ideation among youth in sub-Saharan Africa is very limited [2]. As such, this study contributes to the growing body of literature exploring risk factors for suicidal ideation among youth living in sub-Saharan Africa. Additionally, empirical research on suicidal ideation among street youth and youth living in the slums in developing nations is also scarce [5], despite the growth of urban areas and slums. Moreover, while there has been a previous study on suicidal ideation among youth in the slums of Kampala, the current study expands on previous research by examining suicidal ideation in a larger, more representative sample of youth, with a narrower age range and with a higher proportion of males. Moreover, the current study also examined previously unassessed risk factors [5]. 

Suicide prevention initiatives and mental health treatment interventions are urgently warranted to ameliorate the high prevalence of suicidal ideation and the broad range of adverse life events in this vulnerable population. Previous research has demonstrated a reduction in suicide attempts when a multi-level suicide prevention campaign was implemented [36,37]. Multi-level prevention programs include targeting high-risk adolescents and training youth on self-help activities and coping skills, training and equipping community leaders on suicide prevention tools, and implementing a widespread suicide awareness and prevention campaign [36,37]. Additionally, “connectedness” is an important protective factor against suicidal behavior among adolescents, and the integration of “connectedness” into suicide prevention initiatives is highly recommend [37]. “Connectedness” is commonly defined as social support, social integration [38], and satisfaction with the adolescents’ relationships and environmental factors [39]. These types of interventions may be adapted for implementation in these low-resource settings and to focus specifically on mitigating and modifiable risk factors. However, this is an important area for future research to address. Moreover, clinics and community-based organizations could also screen for suicidal ideation among youth at high-risk as a result of adverse childhood experiences, problem drinking, or other associated risk factors. This is another critically important area for future research to examine the feasibility of implementing screening tools, referral practices, and options for psychosocial support and services in these low-resource settings. 

Several recent studies have documented a high prevalence of stigma and discrimination against people living with mental illnesses in certain countries in Africa, specifically Malawi [40], Ethiopia [41], and South Africa [42]. Because stigma and discrimination against persons with mental illness are highly associated with suicidal risk [43], awareness campaigns may be implemented which aim at eliminating stigma and discrimination associated with mental illness. Inaccurate perceptions of mental illness (e.g., believing supernatural forces are the cause of mental illness [44]) may exacerbate stigma and discrimination against people with mental illness. Awareness campaigns could also focus on addressing these misunderstandings in order to decrease stigma and discrimination.

Future research should also investigate risk factors for suicidal ideation among youth in a longitudinal cohort to better elucidate the relative timing and intervention points for addressing mental health concerns including suicidal ideation to understand and support help-seeking behaviors, use of coping strategies, and other factors that may buffer against the adversity these youth experience while living in the slums. Additionally, it would be important to also investigate other potentially protective factors that may contribute to resiliency among these youth living in the slums and that buffer against the risk of suicidal ideation. Finally, future research should also include more details about the mental health concerns these youth experience, including the use of alcohol and other drugs as a strategy for self-medication.

## 5. Conclusions

This study found that nearly 1/4 of youth living in the slums of Kampala experienced suicidal ideation in the past year. These findings are dire in the context of limited mental health services, treatment options, and high levels of stigma related to mental health issues. Moreover, suicidal ideation was associated with problem drinking and a range of adverse childhood experiences such as physical abuse, orphanhood, homelessness, and rape. Because a population with high levels of suicidal ideation may be less receptive to health promotion strategies, providing the mental health services these youth need is a critically important public health priority. As has been stated and recommended previously [5], providing more mental health services to these youth needs to be viewed as complimentary to prevention efforts underway to address other pressing health concerns. It is our hope that these empirical findings lend support for more research on the mental and other health needs in this vulnerable population that can inform prevention initiatives to be developed and tailored to the specific complex needs of youth living in the slums of Kampala and in similar low-resource settings. 

## Figures and Tables

**Table 1:** Characteristics of youth living in the Slums of Kampala who report suicidal ideation (*n* = 1130).

Demographic and Psychosocial Variables	Suicidal Ideation—Yes	Suicidal Ideation—No	Total
	*n* = 266 (23.54%)	*n* = 864 (76.46%)	*n* = 1130
Gender			
Male	94 (35.33%)	400 (46.30%)	494 (43.76%)
Female	172 (64.66%)	463 (53.70%)	635 (56.24%)
Age, median (IQR)	17 (3)	17 (3)	17 (3)
Education			
Less than primary	95 (35.71%)	301 (34.84%)	396 (35.48%)
Completed primary	58 (21.80%)	205 (23.73%)	263 (23.57%)
Secondary or higher	108 (42.49%)	349 (40.39%)	457 (40.95%)
Ever lived on streets			
Yes	105 (39.47%)	143 (16.57%)	248 (21.97%)
No	161 (60.53%)	720 (83.33%)	881 (78.03%)
Parental living status			
Both parents living	71 (26.69%)	386 (44.68%)	457 (40.44%)
One parent dead	117 (43.98%)	306 (35.42%)	423 (37.43%)
Both parents dead	78 (29.33%)	172 (19.91%)	250 (22.12%)
Alcohol Use (CAGE scores)			
Problem drinking	70 (26.32%)	94 (10.88%)	786 (69.56%)
No problem drinking	58 (21.80%)	122 (14.12%)	180 (15.93%)
Non-drinker	138 (51.88%)	648 (75.00%)	164 (14.51%)
HIV infection			
Yes	37 (13.91%)	79 (9.14%)	116 (10.52%)
No	223 (83.83%)	764 (88.43%)	987 (89.48%)
STI Infection			
Yes	142 (53.38%)	277 (32.06%)	419 (37.08%)
No	124 (46.62%)	587 (67.94%)	711 (62.92%)
Ever been raped			
Yes	77 (28.95%)	114 (13.19%)	191 (16.92%)
No	189 (71.05%)	749 (86.69%)	938 (83.08%)
Physical abuse			
Yes	137 (51.50%)	243 (28.13%)	380 (33.66%)
No	129 (48.50%)	620 (71.76%)	749 (66.34%)

**Table 2:** Demographic and psychosocial characteristics associated with suicidal ideation among youth living in the Slums of Kampala.

Demographic and Psychosocial Variables	Unadjusted OR (95% CI)	Adjusted OR (95% CI)
Gender		
Male	1.00	1.00
Female	1.58 (1.19, 2.10)	1.61 (1.15, 2.25)
Age	1.12 (1.03, 1.22)	0.99 (0.90, 1.10)
Education		
Less than primary	1.00	1.00
Completed primary	0.90 (0.62, 1.30)	1.07 (0.70, 1.61)
Secondary or higher	0.98 (0.72, 1.35)	1.15 (0.78, 1.68)
Ever lived on streets		
Yes	3.28 (2.42, 4.45)	2.65 (1.86, 3.79)
No	1.00	1.00
Parental living status		
Both parents living	1.00	1.00
One parent dead	2.08 (1.49, 2.89)	1.51 (1.05, 2.18)
Both parents dead	2.47 (1.71, 3.56)	1.55 (1.03, 2.35)
Alcohol Use (CAGE scores)		
Problem drinking	3.50 (2.44, 5.01)	1.83 (1.19, 2.80)
No problem drinking	2.23 (1.55, 3.21)	1.33 (0.88, 2.02)
Non-drinker	1.00	1.00
HIV infection		
Yes	1.61 (1.06, 2.44)	0.97 (0.60, 1.58)
No	1.00	1.00
STI Infection		
Yes	2.43 (1.83, 3.21)	1.59 (1.14, 2.21)
No	1.00	1.00
Ever been raped		
Yes	2.68 (1.92, 3.72)	1.49 (1.01, 2.20)
No	1.00	1.00
Physical abuse		
Yes	2.71 (2.04, 3.59)	2.40 (1.75, 3.27)
No	1.00	1.00
